# STepped exercise program for patients with knee OsteoArthritis (STEP-KOA): protocol for a randomized controlled trial

**DOI:** 10.1186/s12891-019-2627-8

**Published:** 2019-05-28

**Authors:** Kelli D. Allen, Dennis Bongiorni, Kevin Caves, Cynthia J. Coffman, Theresa A. Floegel, Heather M. Greysen, Katherine S. Hall, Bryan Heiderscheit, Helen M. Hoenig, Kim M. Huffman, Miriam C. Morey, Shalini Ramasunder, Herbert Severson, Battista Smith, Courtney Van Houtven, Sandra Woolson

**Affiliations:** 1 Center of Innovation to Accelerate Discovery and Practice Transformation, Durham VA Healthcare System HSRD (152), 508 Fulton Street, Durham, NC 27705 USA; 2 Department of Medicine & Thurston Arthritis Research Center, University of North Carolina at Chapel Hill, Chapel Hill, USA; 3 Physical Medicine & Rehabilitation Service, Durham VA Healthcare System, Durham, USA; 4 Department of Biomedical Engineering, Duke University, Durham, USA; 5 Department of Biostatistics and Bioinformatics, Duke University Medical Center, Durham, USA; 6 College of Nursing, East Carolina University, Greenville, USA; 7 University of Pennsylvania, School of Nursing, Philadelphia, PA USA; 8 Department of Medicine, Division of Geriatrics, Duke University, Durham, USA; 9 Claude D Pepper Older Americans Independence Center, Duke University, Durham, USA; 10 Geriatric Research, Education and Clinical Center, Durham VA Healthcare System, Durham, USA; 11 Department of Orthopedics and Rehabilitation, University of Wisconsin, Madison, USA; 12 Department of Medicine, Division of Rheumatology, Duke University, Durham, USA; 13 Orthopedic Surgery Service, Durham VA Healthcare System, Durham, USA; 14 Oregon Research Institute, Eugene, OR USA; 15 Department of Population Health Sciences, Duke University School of Medicine, Durham, USA

**Keywords:** Osteoarthritis, Knee, Exercise, Physical therapy

## Abstract

**Background:**

Physical therapy (PT) and other exercise-based interventions are core components of care for knee osteoarthritis (OA), but both are underutilized, and some patients have limited access to PT services. This clinical trial is examining a STepped Exercise Program for patients with Knee OsteoArthritis (STEP-KOA). This model of care can help to tailor exercise-based interventions to patient needs and also conserve higher resource services (such as PT) for patients who do not make clinically relevant improvements after receiving less costly interventions.

**Methods / Design:**

Step-KOA is a randomized trial of 345 patients with symptomatic knee OA from two Department of Veterans Affairs sites. Participants are randomized to STEP-KOA and Arthritis Education (AE) Control groups with a 2:1 ratio, respectively. STEP-KOA begins with 3 months of access to an internet-based exercise program (Step 1). Participants not meeting response criteria for clinically meaningful improvement in pain and function after Step 1 progress to Step 2, which involves bi-weekly physical activity coaching calls for 3 months. Participants not meeting response criteria after Step 2 progress to in-person PT visits (Step 3). Outcomes will be assessed at baseline, 3, 6 and 9 months (primary outcome time point). The primary outcome is the Western Ontario and McMasters Universities Osteoarthritis Index (WOMAC), and secondary outcomes are objective measures of physical function. Linear mixed models will compare outcomes between the STEP-KOA and AE control groups at follow-up. We will also evaluate patient characteristics associated with treatment response and conduct a cost-effectiveness analysis of STEP-KOA.

**Discussion:**

STEP-KOA is a novel, efficient and patient-centered approach to delivering exercise-based interventions to patients with knee OA, one of the most prevalent and disabling health conditions. This trial will provide information on the effectiveness of STEP-KOA as a novel potential model of care for treatment of OA.

**Trial Registration:**

Clinicaltrials.gov, NCT02653768 (STepped Exercise Program for Knee OsteoArthritis (STEP-KOA)), Registered January 12, 2016.

**Electronic supplementary material:**

The online version of this article (10.1186/s12891-019-2627-8) contains supplementary material, which is available to authorized users.

## Background

Osteoarthritis (OA) is one of the most prevalent chronic conditions in the U.S. Knee OA is particularly common, with one study indicating a lifetime risk of 45% [1, 2]. The prevalence of knee OA is expected to rise dramatically over the next several decades [3]. OA is associated with significant pain, functional limitations, and reduced health-related quality of life [4]. U.S. military Veterans experience a disproportionate burden of OA [5, 6], likely due in part to high rates of joint injuries and loading. One study showed that rates of OA in military service members are about twice as high as those in comparable age groups in the general population [6]. Data from a national survey showed that 32% of Veterans reported a doctor’s diagnosis of arthritis (with knee OA being the most common form), compared with 22% of non-Veterans [7]. Veterans who receive care within the Department of Veterans Affairs (VA) healthcare system have a particularly high burden of OA. For example, national survey data also show that VA health care users are more likely to report a diagnosis of arthritis (43% vs. 30%, *p* < 0.001) compared to Veterans who receive care outside the VA health care system. Furthermore, among Veterans with arthritis (most commonly OA), those receiving VA care are more likely to report limitation in their daily activities because of joint symptoms (63% vs. 42%, *p* < 0.001) [7].

OA treatment guidelines consistently recommend both exercise programs and physical therapy (PT) as core components of managing knee OA [8–10], based on strong evidence for their effectiveness [11–14]. Effect sizes for improvements in pain and function following exercise-based programs for knee OA are comparable to those observed for pharmacological treatment of OA [11]. However, despite the evidence for exercise programs and PT in managing OA symptoms, both are substantially under-utilized [15, 16]. In a study of adults who had or were at risk for knee OA, only 2% of African Americans and 13% of whites were currently meeting physical activity recommendations [16]. In our recent study of VA health care users with knee OA [17], less than half had ever received PT, despite a relatively long average duration of disease (14 years). Although the reasons for low use of PT for knee OA have not been fully elucidated, there are two likely contributors. First, neither treatment guidelines nor prior studies indicate which patients with knee OA have the greatest need for or may benefit most from PT visits (versus lower resource approaches to enhance physical activity) [8–10]. The lack of evidence in this area leaves primary care providers without guidance for making appropriate referrals. Second, outpatient PT visits are a limited resource in many healthcare systems [18], including the VA. Because of these challenges, there is a need to identify strategies to efficiently and appropriately focus PT services and identify complementary, lower resource strategies to help improve physical activity and associated outcomes among patients with knee OA.

This study is evaluating a STepped Exercise Program for patients with Knee OsteoArthritis (STEP-KOA). Stepped care interventions, which begin with low intensity / low resource treatments and “step up” to more intensive treatments if patients do not make clinically relevant improvement, are attractive from both patient and resource allocation perspectives [19, 20] and have a strong evidence base in the context of pain management and other health conditions [21–25]. However, to our knowledge, no studies have previously examined a stepped approach to increasing physical activity for patients with OA. We believe a stepped approach is particularly appropriate in this context for several reasons. First, knee OA is highly common among Veterans, and there is a need for an efficient approach to fostering physical activity in this large group. Second, among patients with knee OA there is considerable variability in pain severity, physical function, exercise abilities, and other factors that likely influence the intensiveness of physical activity intervention needed. A stepped care approach can address this heterogeneity. Third, the demand for outpatient PT for knee OA is increasing, including within the VA, due to the rising prevalence of this health condition. A stepped intervention could guide more focused use of these PT visits for knee OA. As described in detail below, STEP-KOA begins with a low-resource intervention, involving access to an internet-based exercise program for knee OA (Step 1). Participants who do not meet response criteria for pain and function, established by the Outcome Measures in Rheumatology group and the Osteoarthritis Research Society International (OMERACT-OARSI [26]), after Step 1 progress to a more intensive intervention approach, adding telephone coaching to address barriers to physical activity (Step 2). Patients who still do not meet response criteria after Step 2 progress to a more intensive Step 3, involving in-person PT visits. This manuscript describes the protocol for a clinical trial testing the effectiveness of STEP-KOA within the VA healthcare system. Specific aims and associated hypotheses for this project are:

- Aim 1: To examine the effectiveness of STEP-KOA on key patient-centered outcomes among Veterans with symptomatic knee OA.
- *Hypothesis 1:* Veterans who receive STEP-KOA will have clinically relevant improvements in self-reported pain, stiffness, and function, measured by the Western Ontario and McMasters Universities Osteoarthritis Index (WOMAC), immediately following the 9-month program, compared with Veterans in an Arthritis Education (AE) control group.
- Specific Aim 2: To estimate maintenance effects of STEP-KOA at 15 month follow-up, 6 months following completion of the program.
- *Hypothesis 2: At 15-months,* Veterans in the STEP-KOA group will maintain improvements in WOMAC scores achieved at 9 months (i.e. there will be no estimated mean difference in WOMAC scores between 9 and 15 months).
- Specific Aim 3: To describe patients who are non-responders at each Step in the STEP-KOA group, and to examine patient characteristics associated with non-response.
- Specific Aim 4: To examine the cost effectiveness of overall STEP-KOA intervention, compared with the AE control group.

## Methods

This study was reviewed and approved by the Institutional Review Board of the Durham VA Healthcare System (#01933).

### Study sites and design

**Fig. 1:**
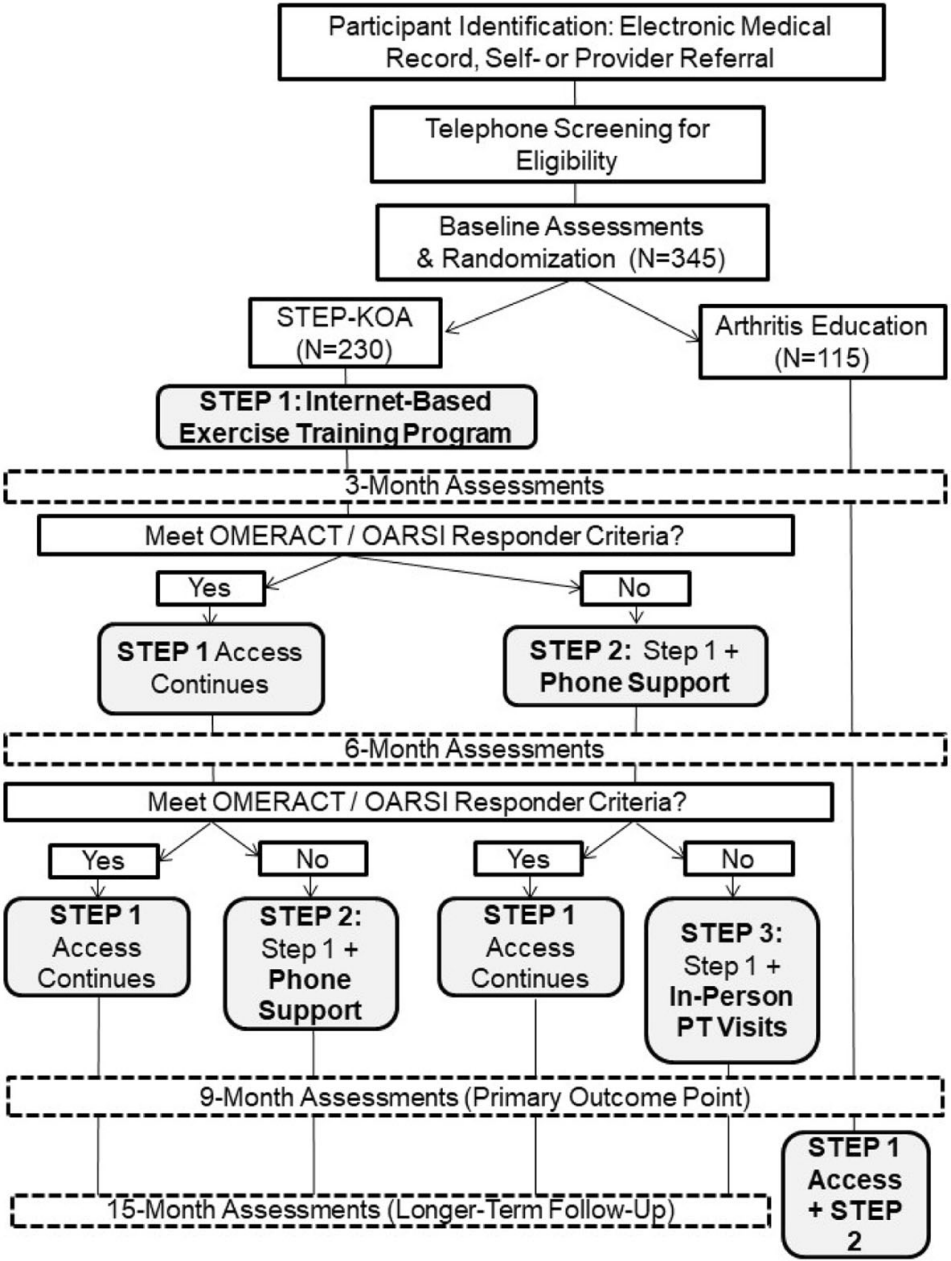
Study Design

This study is being conducted in two sites within the VA Mid-Atlantic Health Care Network, Durham, NC and Greenville, NC. This is a randomized controlled trial with participants assigned to two groups: STEP-KOA and Arthritis Education (AE) (Fig. 1). Participants are randomized in a 2:1 ratio (STEP-KOA: AE Control); this design gives ample sample size to characterize the STEP-KOA process and to explore characteristics of responders and non-responders at each evaluation time point (Specific Aim #3). Randomization is stratified by gender and study site to ensure groups are balanced in these respects. Three and six-month assessments will be used to determine whether participants in STEP-KOA need to progress to more intensive steps. The primary outcome point (Aim 1) will be at 9 months. In addition, Aim 2 will examine whether any effects are maintained in the STEP-KOA group in the 6 months following the intervention period (15 months post-randomization). Following completion of 9-month assessments, participants assigned to the AE control group will be offered access to the internet-based exercise training program, along with the Step 2 exercise coaching calls. All participants are permitted to continue other usual medical care for OA during the full study period.

### Participant eligibility criteria

This study involves *n* = 345 Veterans with symptomatic knee OA. All participants must meet the following criteria: 1) Diagnosis of Knee OA (identified from VA electronic medical records and self-report) and 2) Current Joint Symptoms (based on self-report). Specifically, participants must self-report having an average knee pain of 3 or greater (on a scale of 0–10) over that past 2 weeks. Exclusion criteria are listed in Table 1.

**Table 1:** Exclusion criteria

Currently completing PT visits for knee OA	
Gout (in knee)	
Rheumatoid arthritis, fibromyalgia, or other systemic rheumatic disease	
Dementia, psychosis or active substance abuse disorder	
Meniscus or knee ligament tear in the past 6 months	
Total joint replacement or other major lower extremity surgery in the past 6 months or planned in the next 9 months	
Severe hearing or visual impairment	
Serious / terminal illness	
Hospitalization for a cardiovascular condition in the past 3 months	
Unstable angina	
History of ventricular tachycardia	
Unstable chronic obstructive pulmonary disease (two hospitalizations within the previous 12 months and/or on oxygen)	
Uncontrolled hypertension (diastolic blood pressure > 110 mm/Hg or systolic > 200 mm/Hg, measured at the baseline visit)	
Stroke with moderate to severe aphasia	
Recent history of three or more falls	
Resident of a long-term care facility	
Other health problem that would prohibit participation in the study and/or warrant immediate PT	
Current participation in another OA intervention study	

### Recruitment, enrollment and randomization procedures

Recruitment methods include: 1) direct contact by the study team (primary method), *2) self-referral, 3.) provider referral. Direct contact* of patients by the study team begins with a pull of VA electronic medical record data to identify patients with diagnosis codes for knee OA and no exclusionary diagnoses, followed by an introductory letter. *Self-referral* involves Veterans responding to posters and brochures placed in clinic waiting rooms and other common areas. To facilitate *provider referral*, VA clinicians may give brochures to patients or utilize a consult within the medical record system. For participants identified via all three methods, a screening telephone call is used to further assess eligibility. Patients meeting screening criteria are asked to come to their VA site for an initial visit, which consists of consent and baseline assessments.

The randomization sequence is generated by a study statistician and maintained in the tracking database. The randomization assignment for each participant can only be obtained by a study team member once that participant has completed consent and baseline assessments and is ready to be notified of their group assignment. After the enrollment visit, at study team member calls participants to give them their randomization assignment. At that time, participants assigned to STEP-KOA are given an orientation to the Step 1 website and assistance with getting set up as a new user. Participants are also sent information on how to use the website, along with general information about physical activity and osteoarthritis. Participants are also sent a study-issued iPad if needed.

### STEP-KOA intervention

#### Overview

Components of the STEP-KOA program are evidence-based and focus on enhancing physical activity but differ in the amount, type and mode of care provided. Participants begin with the intervention component requiring the least resources to deliver (access to a tailored, internet-based exercise training program) and progress to additional components if they fail to meet response criteria for clinically relevant improvement in pain and / or function. Participants are evaluated for treatment response every 3 months, based on prior research indicating this is an adequate time period to observe meaningful changes in pain and function [27]. All participants in STEP-KOA will continue to have access to the Step 1 internet-based program for the full 9-month intervention period, regardless of whether they progress to Steps 2 and 3. This is because the internet-based program may serve as a complementary resource to participants who go on to other steps, and this approach is similar to other stepped care interventions [21, 23]. Some participants who initially meet response criteria for improvement at the first (3-month) assessment point (and therefore remain at Step 1) may regress by 6 months and no longer meet benchmarks when compared to baseline pain and function. These participants are then advanced to the Step 2 intervention at the 6-month time point; this approach is also similar to other stepped care interventions [21, 23].

The OMERACT-OARSI responder criteria are used to determine whether participants progress to Step 2 and Step 3 interventions [26]. These criteria were established using a combined data-driven and expert opinion approach, and they have been used in over 30 clinical trials of behavioral, pharmacological and surgical interventions for OA [2, 28]. Participants can meet OMERACT-OARSI response criteria in two ways: **1)** ≥ 50% improvement in pain OR function AND absolute change ≥20, **2)** Improvement in at least two of the following: pain ≥20% and absolute change ≥10; function pain ≥20% and absolute change ≥10; patient’s global assessment ≥20% and absolute change ≥10. The project coordinator, not blinded to study assignment, informs participants via telephone about whether they progress to Step 2 and 3, following completion of 3- and 6-month assessments.

#### Step 1: Internet-based Exercise Training (IBET)

The IBET program used for Step 1, described in detail previously, was developed by a multidisciplinary team of patients and clinicians. The program was designed with an aim of mirroring a “real life” rehabilitation experience for patients with knee OA, including provision of personalized exercise recommendations, monitoring and progression of activities [29]. Results of a pre-post pilot study showed that the modified short form of the Western Ontario and McMaster Universities Osteoarthritis Index (mSF-WOMAC, a measure of lower extremity pain, stiffness and function [30]) decreased by about 7 points following the program, which is a large effect size [29].

The program is comprised of the following components:

1. Tailored Exercise Routines. The program includes five exercise levels that span a continuum of functional abilities. Initial assignment to an exercise level is based on patients’ responses to the mSF-WOMAC and other items assessing exercise abilities and function. The algorithm for assigning exercise levels was validated against recommended assignments by physical therapists familiar with the exercise levels. Exercises within each level, including strengthening and stretching, are based on clinical guidelines and selection by a panel of orthopedic surgeons, physiatrists, rheumatologists and physical therapists. Exercises emphasize the lower body. Specific exercises included in a participant’s assigned routine are randomly selected from within the appropriate level, and participants may request to change the specific exercises within a level at any time. Participants are also prescribed aerobic exercises appropriate for each level.
2. Animations of Strengthening and Stretching Exercises (Fig. 2). When participants log in to the program, a static version of their strengthening and stretching exercises is displayed. Patients can click on each exercise to view the animated version. These video presentations are important for instructing patients in safe, appropriate ways to perform each exercise.

**Fig. 2:**
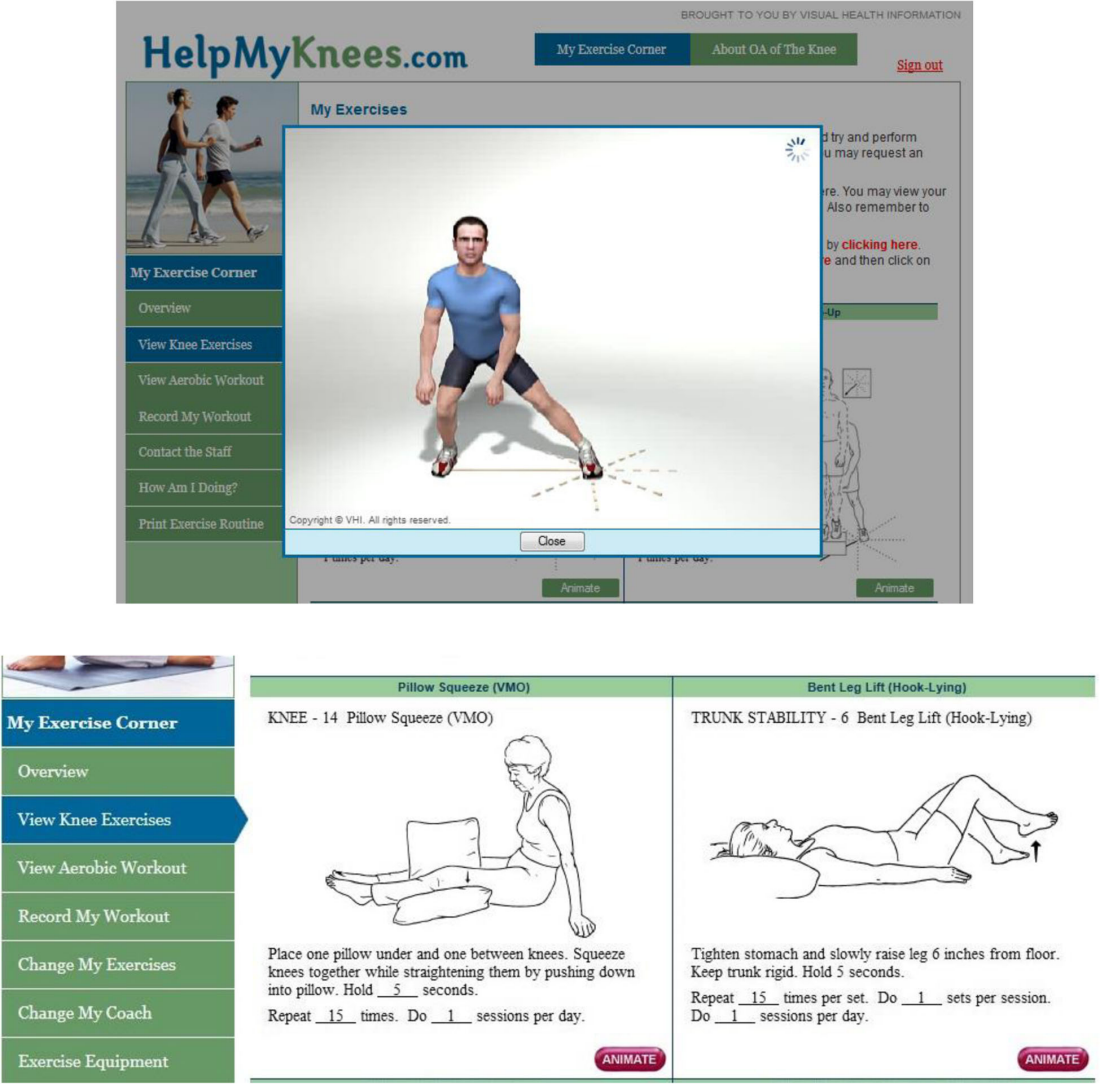
Sample Screen Shots from the Internet-Based Exercise Training Program (Used with permission from Visual Health Information (VHI)
3. Monitoring and Progression of Exercise Routine. Participants are asked to record their exercises after each session. Participants can request at any time to move to a more difficult or easier exercise level, but they are only enabled to move to a harder exercise level if their mSF-WOMAC score is better than or equal to their previous score. If this score is worse than previously, they are automatically given a new exercise routine at their current level. With increasing exercise level, the difficulty of the routine progresses based on a combination of number of assigned strengthening / stretching exercises, difficulty of the specific exercises, and resistance of the strengthening exercises (e.g., body weight, amount of weight in the ankle weights); duration of recommended aerobic activity also increases with each level.
4. Pain Monitoring. Patients are also asked to report any increases in pain as a result of their exercises. If patients record an increase in pain after three consecutive sessions but do not request a lower exercise level, a suggestion is given to consider trying a lower exercise level. If patients do not report increased pain for 2 weeks, they are encouraged to try a more difficult level.

Participants in this study are given an individualized code and instructions for accessing the IBET program. They are also given weigh-t adjustable ankle weights and elastic resistance bands, since these are utilized in some exercises. If participants do not enter the website within 2 weeks of being provided access, they receive a call from a project coordinator, encouraging them to access the website and inquiring about any technical difficulties. Participants are given a telephone number to contact the project coordinator if they need additional technical support regarding the website or study-issued iPad. Participants are encouraged to access the IBET program immediately after being randomized and throughout the study period. Based on physical activity guidelines [31], we recommend that participants perform stretching and strengthening exercises at least three times per week and aerobic exercises daily (or as often as possible), guided by the website.

#### Step 2: Telephone-Based Physical Activity Coaching

Participants who move to Step 2 receive bi-weekly telephone calls from a physical activity coach for a 3-month period. This component was chosen because studies indicate that personal support from an physical activity coach (or similar role) can increase physical activity [32]. Also, although this approach is more resource intensive than an internet-based program, it is less costly than in-person PT visits and does not require Veterans to travel to a VA facility.

The overall goals of Step 2 calls are to address any OA or health-related difficulties participants are having with their exercise program, provide additional social support for physical activity, and reinforce information about the benefits of physical activity. Each phone call is guided by a script. Content for the first telephone call includes: 1) Introduction of the physical activity coach and their role. 2) Questions for the participant about their OA symptoms, health problems, or other barriers that present challenges to their engagement in activity, 3)_Review of guidelines for physical activity for individuals with OA including description of “safe” exercises, general instructions for performing aerobic, strengthening and stretching exercises, good long-term goals for each type of exercises (150 min of aerobic exercises weekly, strengthening exercises 2–3 times per week, stretching daily), and tips for managing pain with physical activity. 4) Description of SMART (Specific, Measurable, Action-oriented, and Time-Bound) [33] goal setting, 5) Goal-setting for the next 2 weeks, 6) Time for additional questions from the participant. For calls #2-#5, the content includes: 1) Asking the participant about their physical activity about the prior call, including what they have been doing, any barriers they faced and strategies to resolve, and any increases in joint pain, 2) Discussion of other potential strategies to address any barriers the participant is facing regarding their physical activity, 3) Goal-setting for the next 2 weeks, 4) Time for additional questions from the participant. The content for call #5 includes topics included for calls #2-#5, as well as: 1) Review of the participant’s progress toward SMART goals since the beginning of the study and encouragement of the participant for their progress, 2) Planning for future physical activity progression and dealing with setbacks.

Motivational interviewing strategies, including use of open-ended questions and reflective listening, are employed to identify any ambivalence patients experience about engaging in physical activity [34]. All physical activity coaches (*n* = 3) received training from co-investigators with experience in exercise science, physical activity coaching, telephone-based interventions and motivational interviewing (Allen, Hall, Morey). Physical activity coaches performed calls with “mock” participants prior to intervention delivery, with co-investigators providing feedback. For each coach, a subset of intervention calls are audio-recorded, and one or more co-investigators listens to the call and provides specific feedback using a standardized fidelity checklist. Intervention calls were also recorded in a study database, which assisted with scheduling logistics as well as documenting participants’ goals so that the coaches could refer to these in subsequent calls.

#### Step 3: PT Visits

This intervention component was chosen because physical therapists have specialized training to evaluate functional impairments and biomechanical issues and can assist patients with tailoring exercises to address these deficits. Physical therapists can also evaluate patients’ needs for knee braces, shoe lifts, or other assistive devices. We posited that patients who do not experience meaningful improvement after remotely delivered physical activity interventions (Steps 1 and 2) may have greater functional impairments or other underlying clinical issues that will benefit from in-person attention by a physical therapist.

The Step 3 intervention is based on usual PT care for knee OA. Specifically, core components of PT for knee OA include: instruction in a tailored exercise program, instruction in activity pacing and joint protection, and evaluation of mobility, stability, function, knee alignment, limb length inequalities, specific areas of weakness or inflexibility, and need for mobility aids, knee braces, and shoe orthotics. Participants are encouraged to attend between 3 and 7 1-on-1 PT visits, based on progress toward goals. The first PT session lasts about 1 h, since this involves an initial evaluation, and remaining visits are 30 min each. To mirror VA processes for providing travel-related compensation for patients, study participants are paid $10 for each physical therapy visit, plus an additional amount that varies by distance traveled.

The first session for Step 3 intervention begins with an evaluation that was developed in collaboration with VA physical therapist to reflect usual care. Specific components of the evaluation include: 1) Assessment of participants’ knee symptoms, 2) Assessment of prior injuries and falls, 3) Discussion of participants’ goals for therapy, 4) Palpation to check for edema, warmth, tenderness, crepitus and bony enlargement, 5) Evaluation of strength and active range of motion for knee flexion, knee extension, ankle plantar flexion and ankle dorsiflexion, 6) Assessment of current knee pain severity, 7) Knee alignment (varus, valgus, neutral), 8) Check for leg length inequality, 9) Evaluation of static balance, dynamic balance and gait problems 10) Additional tests as indicated and at the physical therapists discretion (for example, muscle length abnormalities, tests for anterior and medial cruciate ligament abnormalities). Following this evaluation the physical therapist discusses treatment recommendations and follow-up plans with the participant, which could include manual therapy, balance/ neuromuscular education, balance / neuromuscular education, gait / stair training, and referrals for additional evaluations for assistive devices, knee braces, and shoe lifts / orthotics. The final component of the first visit involves review of the participant’s current exercise and providing recommendations for tailoring or modifications to their home exercise program. Although the physical therapists tailor participants’ home exercise programs to their functional abilities and goals, general recommendations are that the program should include about 4–5 exercises and take about 15 min to complete each time it is performed.

At all subsequent PT visits begin with a brief re-assessment of pain and home exercise. This is followed modifications and progressions to the home exercise program and having the participant perform the new exercise set to ensure appropriate performance. The physical therapists may also follow up on previous treatment recommendations (e.g., referrals for knee braces, manual therapy). All PT visits are documented in a study database.

### Arthritis education (AE) control condition

The AE control group was selected for two reasons. First, as this is an active condition (rather than a control condition with no intervention), it allows participants to receive an OA-focused intervention immediately. This can help to improve satisfaction and retention participants, and it helps to account for any effects due simply to receiving an intervention (e.g., attention effects). Second, AE has been used as an effective, feasible control condition in prior studies of behavioral interventions for OA [35–37]. Participants in the AE control group receive low literacy educational materials via mail every 2 weeks for 9 months. Because STEP-KOA is a multi-component intervention, with participants receiving different numbers of Steps, it is not feasible to implement a control condition that mirrored the exact intervention “dose” and contact type received by all participants in the intervention group. However, the AE condition achieves the goal of providing an active, OA-related control condition. We selected a mail format because it mirrors the “remote” aspect of the Step 1 intervention. We selected the 2 week interval for mailings because it mirrors aspects of each of the STEP-KOA components: The AE intervention includes a comprehensive set of topics related to OA and its management, based on established treatment guidelines (Table 2) [8, 38]. All AE materials are shown in Additional file 1.

**Table 2:** Topics for AE intervention

What is OA?	
Diagnosis of OA	
Risk Factors for OA	
Health Care Providers And OA	
Pain Medications (Oral)	
Topical Pain Medications	
Knee Braces	
Shoes and Orthotics	
Physical Activity	
Weight Management	
Joint Protection	
Pain Coping Skills	
Surgery	
Complementary and Alternative Therapies	
OA and Sleep	
OA and Mental Health	
OA and Fatigue	

### Measures

Study assessments are conducted in person at baseline and 9-months (primary outcome point). Interim assessments (3-months, 6-months) and longer-term follow-up (15-months) are conducted via telephone. Assessments are conducted by a research assistant blinded to group assignment. Participants’ responses are entered into a study database (DatStat Illume®) that was programmed to minimize risks of out-of-range and missing data. The study measures database and tracking database are housed on a secure VA server, accessible only to authorized study team members. Participants are paid $40 for in-person and $20 for telephone-based assessments.

#### Primary Outcome: Total WOMAC Score (Collected at Baseline and all Follow-Up Time Points)

The primary outcome measure is the WOMAC, a measure of lower extremity pain (5 items), stiffness (2 items), and function (17 items), with items rated on a Likert scale of 0 (no symptoms) to 4 (extreme symptoms). The reliability and validity of the WOMAC total score and subscales have been confirmed [39]. The WOMAC has been widely used in trials of behavioral interventions for knee OA, confirming its sensitivity to change. The WOMAC has also been validated for use via telephone [40].

#### Secondary Outcome: Objective Physical Function (Collected at Baseline and 9-Month Follow-Up)

Physical function assessments are based on OARSI recommendations for clinical trials of knee OA [41]. These tests have been previously validated and have shown good sensitivity to change in clinical trials among patients with OA. Tests include a 30 s stair stand test, a 40 m fast-paced walk, a timed get up and go test, stair climbing test, and a 6-min walk test, following previously established procedures for each. The 30 s stair stand asks participants to rise and sit back down in a chair as many times as they can during that time period, without using hands or arms for support [42]. The 40 m fast-paced walk is a timed test of walking twice back and forth (as fast as participants are able) over a 10 m distance [43].The timed get up-and-go test requires the participants to stand from a standard arm chair, walk 3 m and then return to sitting in the same chair (as quickly and safely as possible). The stair climbing test measures the time it takes to ascend and descend a flight of 12 steps (each step 18 cm high and 28 cm deep). Participants are asked to complete the test as quickly as they feel safe and comfortable. The use of one handrail is allowed if necessary, but patients are encouraged to minimize their use of the handrail. The 6-min walk test is a self-paced task during which individuals are instructed to walk as far as they can during a 6-min period. Walking aids are permitted as needed. Following each tests, participants are asked to indicate the maximum pain they experienced, on a scale of 0–10, during the test.

### Exploratory and process measures

#### Self-Efficacy for Exercise Scale (Collected at Baseline and 9-Month Follow-Up)

The Self-Efficacy for Exercise scale assesses individuals’ confidence in engaging in exercise in nine different situations that could present barriers (including having pain when exercising) [44]. For each situation, individuals are asked to rate their confidence in being able to exercise three times a week for 20 min each time, on a scale of 0 (not confident) to 10 (very confident). A total score is derived by taking the average across the 9 items, resulting in a possible total score ranging from 0 to 10. Validity of this measure was confirmed by expected associations with actual exercise, as well as physical and mental health.

#### Social Support for Exercise Scale (Collected at Baseline and 9-Month Follow-Up)

This scale includes 13 items that assess the frequency with which friends and family members (separately) engage in behaviors that may either support exercise (e.g., “Gave me encouragement to stick with my exercise program”) or discourage exercise (e.g., “Complained about the time I spend exercising”) [45]. All items are measured on a scale of 1 (none) to 5 (very often). The scale has shown acceptable test-retest reliability, internal consistency reliability, and concurrent criterion related validity through a strong correlation with exercise habits.

### Physical activity and adherence measures

#### Physical Activity Scale for the Elderly (PASE) (Collected at Baseline and all Follow-Up Time Points)

The PASE is a self-report, 12-item scale that measures occupational, household, and leisure activity during a 1-week period [46]. This scale was developed for use among older adults and is appropriate for patients with knee OA who typically have more limited physical activity than the general population. The PASE has been validated for use via telephone.

#### Additional Self-Report Physical Activity Items (Collected at Baseline and all Follow-Up Time Points)

We are also specifically interested in purposeful exercise behaviors. Participants were asked to report the number of times and minutes per week, on average, they were completing strengthening, stretching, and aerobic exercises.

#### Adherence to Intervention Step Components

Participants’ use of the IBET program is tracked on the website. For participants who advance to Step 2, we collect information on the number of scheduled phone calls completed, and for participants who advance to Step 3, we collect information on the number of PT visits attended.

#### Participant Characteristics (Collected at Baseline only)

We collect the following participant demographic and clinical characteristics: age, race / ethnicity, gender, household financial state, education level, work status marital status, body mass index, questions about internet and technology use, the Patient Activation Measure, comorbid illnesses (Self-Administered Comorbidity Questionnaire) [47], joints with OA, self-rated general health (excellent, very good, or good vs. fair or poor).

#### Knee OA and Related Care During Study Period (Collected at Baseline 9-Month and 15-Month Follow-Up)

We assess use of treatments for knee OA at baseline and follow-up assessments, including pain medications, topical creams, knee braces, joint injections, physical therapy and assistive devices. (At follow-up visits, participants are asked about OA treatment use since their last study visit.) These items are assessed through a combination of self-reported and VA electronic medical records. Participants who start new OA treatments during the study period, but any observed between-group differences in these patterns will be evaluated in exploratory analyses. Because of the drastic improvements typically associated with joint replacement surgery, participants having this surgery during the study period are excluded at that time point.

### Measures for cost-effectiveness analysis

#### Intervention Costs

We will use a micro-costing approach to derive labor and equipment costs for the STEP-KOA intervention. Labor costs for Step 1 include programmer time to maintain the website and phone calls for technical support. Labor costs for the Step 2 intervention include the time needed to train the physical activity coach and to conduct the telephone calls; this time includes any required pre-call preparation, post-call activities, partial call completions, call attempts and callbacks. Labor costs for Step 3 involve the physical therapist’s time to complete in-person visits. Hourly wage + fringe benefit rates for personnel will be applied to the labor time to derive total labor costs. The equipment costs for Step 1 include ankle weight and elastic bands, as well as the Apple iPad Air 16GB with Mobile Broadband Access Calling Plan costs for a subset of participants. Equipment costs for Step 3 include any devices recommended by the physical therapist. Total labor and equipment costs will be divided by the number of patients in the STEP-KOA arm to derive per-patient intervention cost. We note that costs will vary across participants, as participants will not all receive the same Steps. We will report this variability but are primarily interested in the average cost per participant.

#### Patient Resource Utilization and Costs

The intervention may affect primary care and specialist outpatient visits for OA and outpatient pain medication use. Outpatient encounters will be categorized using clinic stop codes of interest, as well as a count of total encounters and total costs of OA-related outpatient care. We will also include fee basis OA-related outpatient care and costs of the same categories because these are of increasing importance to VAs. Our primary analysis will focus on cost categories specific to OA-related outpatient care and pain medications because these were the main types of utilization that we a priori expected the intervention to affect (e.g. reduce utilization in these domains). In a sensitivity analysis, we will aggregate total costs of outpatient encounters and outpatient medications (e.g. not coded as being specifically to address OA-related care), to see if there were spillovers from OA-specific outpatient care and outpatient pain medication, to the outpatient and outpatient pharmacy setting more broadly.

#### Effectiveness Measures

We use two effectiveness measures to calculate cost effectiveness ratios: WOMAC units (described above) and the EuroQoL EQ-5D-5 L questionnaire. The EuroQol health outcome measure (EQ-5D-5 L and EQ-VAS) allows us to calculate quality-adjusted life years (QALYs), and it has been used successfully in previous research among patients with arthritis [48].

#### Participant Feedback on STEP-KOA (Collected at 9-Month Follow-Up Only)

Participants in the STEP-KOA group are asked open-ended questions regarding the intervention components. For example, we will ask participants about usability of the IBET website, appropriateness of the number and length of telephone sessions with the exercise counselor, content of PT visits, and ways we might improve the interventions.

### Data analyses

#### Primary Analysis (Specific Aim 1)

The main study outcome, WOMAC, is a continuous measure collected at baseline, 3, 6, 9 (primary assessment time) and 15 months (STEP-KOA group only). A linear mixed model with an unstructured covariance to address within-patient correlation between repeated measures over time will be used to fit a constrained longitudinal data model, in which baseline WOMAC score is modeled as a dependent variable in conjunction with the constraint of a common baseline mean across treatment arms [49]. We will estimate the parameters in the model and set up contrasts for tests of hypotheses using the SAS procedure MIXED (Cary, NC). For improvement in precision, the model will be adjusted for stratification variables [50].

#### Secondary Analyses

Since the secondary outcomes for objective physical function are continuous, longitudinally collected measures, we will use similar modeling procedures as those described above for WOMAC scores to assess between-group differences at 9-month follow-up. We will also examine Poisson or negative-binomial mixed models as a sensitivity analysis as these outcomes (particularly chair stands) can be skewed and assumption of a normal distribution may not be reasonable.

For Specific Aim 2, to examine the maintenance effects of STEP-KOA at the15-month follow-up, we will add the 15-month outcomes and the 15-month time-point to the fixed effect portion of the model. We will set up contrasts of model parameters to estimate the difference and associated 95% confidence intervals in outcomes between the 9-month and 15-month time points.

For Specific Aim 3, we are interested in understanding the flow of responders and non-responders through the STEP-KOA intervention. We will first calculate proportions of responders and non-responders at each time point and describe responder patterns longitudinally. We will also calculate proportions of individuals who meet response criteria in the two different ways permitted in the OARSI-OMERACT criteria. We will then examine characteristics associated with responder status at each time point, using multivariable logistic regression models. In these models, we will first focus on four a priori patient characteristics: age, baseline pain and function, and baseline self-rated health, to avoid spurious statistical findings [51]. Based on clinical experience, we believe these variables have potential to predict response, and they can be assessed easily and quickly, making them practical screening tools.

#### Missing Data

Because the main predictors of interest, intervention arm and patient characteristics, are collected at baseline, we do not anticipate much missing data in these variables. There may be missing values in the follow-up outcome measures due to dropout, death, a missed interim assessment, or item non-response. Our main analysis technique for the primary outcomes, general linear mixed models via maximum likelihood estimation, implicitly accommodates missingness when missingness is due either to treatment, to prior outcome, or to other baseline covariates included in the model, defined as missing at random [52, 53]Depending on the type and scope of missing data [54], we will also explore multiple imputation as a sensitivity analysis conducted via the SAS procedure PROC MI or the SAS macro IVEware (http://www.isr.umich.edu/src/smp/ive/).

#### Sample Size

The sample size estimate of *n* = 345 patients was based on a 2:1 randomization and the comparison of the primary outcome between the STEP-KOA and AE control arms at 9 months. Since AIM 3 is to describe the responder patterns for the Step progression in STEP-KOA we used a 2:1 randomization to facilitate our ability to evaluate these patterns [55]. For sample size calculations we used methods appropriate for Analysis of Covariance type analyses [56], which are equivalent in terms of efficiency to our linear model in randomized trials [49]. This method is based on performing a two-sample t-test sample size calculation for the between group difference and adjusting based on an assumed correlation between baseline and follow-up time point outcome measures. Based on our previous data, we assumed a correlation of 0.4 between baseline and follow-up WOMAC scores, a standard deviation of 17.5 and an attrition rate of 20% at 9-months [17, 57, 58]. With 80% power, alpha = 0.05, standard deviation = 17.5, rho = .4, and approximately 20% attrition rate by 9-months, 230 and 115 participants are needed in the STEP-KOA and AE groups, respectively, for an effect size of 0.33. This corresponds to a 5.8 point difference in mean total WOMAC scores at 9-months between STEP-KOA and AE, which is a clinically relevant improvement [59]. We are also powered to detect medium effect size differences in secondary study outcomes.

##### Economic Evaluation

The overall goal of Specific Aim #4 is to provide the VA with information about the value of the STEP-KOA program, from a cost effectiveness perspective. In addition to informing the VA regarding the overall average cost of STEP-KOA per patient, this evaluation will provide information on how much improvement in outcomes can be achieved for that cost. The analysis will begin with descriptive statistics of the cost and effectiveness (utility) data. We will examine each measure of effectiveness, WOMAC and mean QALY (and the pain/discomfort sub-core of the EQ-5D-5 L), using the general analytic procedures described above for primary and secondary outcomes. We will calculate the incremental cost effectiveness ratio (ICER) of STEP-KOA compared to the AE control group, separately for WOMAC and QALYs. The ICER will be calculated as the difference in the average total cost per participant STEP-KOA and AE, divided by the difference in the average effectiveness per participant between STEP-KOA and AE. Bootstrapping of estimates, multiple imputation for missing EuroQoL values, and sensitivity analyses will all be conducted to ensure robustness of our results.

## Discussion

This study has several important features. First, it is a novel model for combining exercise-based interventions with knee OA. To our knowledge, prior studies have examined individual components (e.g., PT, home-based exercise) but have not combined them in a stepped approach. This strategy can be cost-saving for health systems like the VA and can help to tailor the intervention approach to patient needs. Second, this study is being conducted in the VA health care system, which is important because of the increased burden of OA in Veterans. The Step 3 intervention is being delivered by VA physical therapists in order to mirror a real-world clinical scenario and usual care approach to PT for patients with knee OA. Third, this study will not only evaluate the effectiveness of STEP KOA but also includes supporting aims that will inform decisions about downstream implementation. These include a cost-effectiveness analysis and exploration of patient characteristics that predict treatment response and the need to transition to more resource intensive steps of the intervention.

We recognize this study also has limitations. First, it is being conducted in two VA healthcare sites, and generalizability may be limited. Second, due to time limitations of the project award period, we will not be able to fully assess longer-term effects of the intervention. Third, this study involves an internet-based intervention component. Although we aimed to enhance generalizability of the study by providing iPads to participants who did not have internet regular access, an internet-based program might not be accessible to all patients in a real-world setting.

In summary, this study is evaluating a novel, stepped approach to providing exercise-based interventions to patients with knee OA. Given the rising prevalence of knee OA, the centrality of exercise as a first-line treatment component, and the need to increase use of exercise-based interventions for patients with knee OA, we believe this study fills an important need. This type of intervention may be particularly relevant for health care settings where there are limitations to PT access and the need for other approaches to provide support for appropriate exercise. Health systems could provide access to an IBET program and telephone-based coaching in lieu of an immediate referral to PT, assess response, then advance to PT only if additional treatment is warranted based on improvement and any unresolved functional issues. The Step 1 and 2 interventions could also be offered at the time of referral to PT when patients have a significant wait time before a visit is scheduled. If this intervention is shown to yield clinically relevant improvements and be cost effective, next steps will involve planning feasible implementation strategies.

## Additional file

Additional file 1: Attention Control Intervention Materials. (PDF 544 kb)

## Abbreviations

AE: Arthritis Education
IBET: Internet-Based Exercise Training
ICER: Incremental Cost Effectiveness Ratio
mSF WOMAC: Modified Short Form of the Western Ontario and McMaster Universities Osteoarthritis Index
OA: Osteoarthritis
OMERACT-OARSI: Outcome Measures in Rheumatology group and the Osteoarthritis Research Society International
PASE: Physical Activity Scale for the Elderly
PT: Physical Therapy
QALYs: Quality-Adjusted Life Years
SMART: Specific, Measurable, Action-oriented, and Time-Bound
STEP-KOA: STepped Exercise Program for patients with Knee OsteoArthritis
VA: Department of Veterans Affairs
WOMAC: Western Ontario and McMasters Universities Osteoarthritis Index
