# Supplementary material for: STepped exercise program for patients with knee OsteoArthritis (STEP-KOA): protocol for a randomized controlled trial
Source: BMC Musculoskelet Disord. 2019 May 28;20:254. doi: 10.1186/s12891-019-2627-8 (PMC6540433; doi:10.1186/s12891-019-2627-8)
Supplement: Supplementary file 1 — Attention Control Intervention Materials. (PDF 544 kb) [file 12891_2019_2627_MOESM1_ESM.pdf]

WHAT IS OSTEOARTHRITIS?

- Osteoarthritis (OA) is the most common joint condition. It affects about 27 million Americans.

- In OA, cartilage wears away.

- Cartilage is the tissue that covers the ends of bones and allows joints to move with ease.

- In later stages of OA, bones can develop growths called "spurs." Also, parts of bone or cartilage may chip off and float around the joint.

- OA can affect any joint. It is most common in the knees, hips, back, neck and hands.

OSTEOARTHRITIS SYMPTOMS can include:

✓ Pain (especially after activity)
✓ Stiffness
✓ Mild swelling
✓ Clicking or cracking
✓ Tenderness
✓ Difficulty with doing some daily activities

HOW IS OSTEOARTHRITIS DIAGNOSED?

A doctor may use the following information to see if you have osteoarthritis (OA):

- Your description of your joint symptoms

- Joint exam
  - How well does the joint move?
  - Is the joint tender, painful or swollen?

- X-Ray
  - Most common test to diagnose OA.
  - Can show joint damage and other changes.

- MRI (Magnetic Resonance Imaging)
  - Can provide a better view of some joint changes.
  - Can sometimes detect OA earlier.

- Joint Fluid Analysis
  - Uses a needle to get fluid from the joint.
  - Can help to rule out infection and other types of arthritis.
  - Used less often than other tests.

Your doctor may repeat these tests to check on your OA from time to time. This can help to see if your joints are changing or if different treatments may be helpful.

RISK FACTORS FOR OSTEOARTHRITIS

We don’t know all of the causes of osteoarthritis (OA). Some things that can increase the risk of getting OA are:

- ✓ Being overweight
- ✓ Past joint injuries
- ✓ Being female
- ✓ Older age
- ✓ Having jobs that involve a lot of knee bending, kneeling, squatting or other joint stress
- ✓ Having knees that bow in (“knock kneed”) or bow out (“bow legged)
- ✓ Having family with OA

BEING OVERWEIGHT and BEING PHYSICALLY INACTIVE can also increase the risk of OA <u>getting worse</u>. These are two factors you <u>can</u> change.

If you need support or advice for losing weight or exercising, ask your doctor about resources that can help you.

HEALTH CARE PROVIDERS AND OSTEOARTHRITIS

Three key types of health care providers are often part of the team for managing osteoarthritis (OA):

Primary Care Providers: usually coordinate OA care and give referrals to other providers and resources.

Physical Therapists: help with exercises, improving function, and devices like knee braces.

Orthopedic Surgeons: give certain types of joint injections, perform joint replacements and other surgeries.

<u>Other providers who can be part of this team are</u>: rheumatologists, occupational therapists, psychologists, social workers, physiatrists, podiatrists, chiropractors, exercise physiologists and massage therapists.

TIPS FOR TALKING WITH YOUR HEALTH CARE PROVIDERS ABOUT OA

- ✓ **Tell your provider if your joint symptoms are getting worse or affecting your daily activities. Don't assume this is "normal aging" that can't be helped.**
- ✓ **Bring a list of questions to your clinic visits.**
- ✓ **As your primary care provider about other clinicians or community resources to help you manage OA.**

PAIN MEDICATIONS AND OSTEOARTHRITIS

There are 3 main types of pain medications for osteoarthritis (OA):

Acetaminophen: This is often the “first line” treatment for mild OA pain. The most common brand is Tylenol.

NSAIDs (Non-steroidal anti-inflammatory drugs): These can be prescription or over-the counter. NSAIDs are the most commonly used medications for OA. They can have side effects like stomach bleeding. Talk with your doctor about these risk if you are taking NSAIDs.

Narcotic or Opioid Analgesics: These are often used for more severe pain or if people cannot take other types of pain medications. These also have risks that you should discuss with your doctor.

Your doctor also may recommend other types of medications to help with OA symptoms.

TIPS FOR OA MEDICATION USE

- ✓ **Tell your health care provider immediately if you are having side effects from a pain medication.**
- ✓ **Tell your provider if you don’t think your pain medications are working well. There may be other choices for you.**
- ✓ **Talk with your doctor about a medication schedule that may help to manage your pain best.**

TOPICAL CREAMS AND OSTEOARTHRITIS

Creams or gels rubbed on the skin around your joints may help with your osteoarthritis (OA) pain. Some types are:

CAPSAICIN: This type of cream can cause a burning feeling on your skin. Some brands are Theragen and Zostrix.

SALICYLATES: These creams contain the same ingredient found in aspirin. Examples are Bengay and Aspercreme.

VOLTAREN GEL: This is a prescription non-steroidal anti-inflammatory gel.

"COUNTER-IRRITANTS": These make your skin feel hot or cold. This can 'over-ride' the feeling of OA pain. Examples are Icy Hot and Biofreeze.

DO CREAMS AND GELS REALLY WORK FOR OA?

- ✓ **We are still learning about how much different creams and gels help knee OA.**
- ✓ **It seems that Capsaicin and Voltaren gel <u>may</u> work better for OA.**
- ✓ **Talk with your doctor about using these. You may want to try different kinds to see what helps you most.**

JOINT INJECTIONS AND OSTEOARTHRITIS

Joint injections are another tool for managing osteoarthritis (OA) pain. There are 2 main types of joint injections:

1.) Steroid (or corticosteroid) Injections: Effects vary across people but often last a few months. You can usually only get these injections a few times per year.

2.) Hyaluronic Acid Injections (like Synvisc): These are typically used after trying other medications and injections. Effects also vary across people. You can usually get these injections about twice per year.

Other types of injections are being studied. These include Platelet Rich Plasma (PRP) and Prolotherapy (sugar water). We still don't know much about how well these other types of injections work for knee OA.

You can ask your doctor about the types of injections that may be best for you.

KNEE BRACES AND OSTEOARTHRITIS

Braces can support your knees and help keep them in line. If your knee feels unstable, a brace could help it to feel steadier. Braces help when you are walking or doing other activity.

There are 3 main types of knee braces used for OA:

Knee Sleeve

Gives some support to the whole knee. You can buy these at a drug store.

Hinged Brace

Gives more support than a knee sleeve. Your doctor or a physical therapist can recommend one of these.

Unloader Brace

These shift the load in your joint. They can take pressure of parts of your knee that are most painful. A physical therapist or other health care provider can fit you with one of these.

If you would like to try a knee brace, or a new type of brace, ask your doctor for advice.

FOOTWEAR AND OSTEOARTHRITIS

Proper shoes are very important for managing knee osteoarthritis (OA).

Here is what we know about shoes and OA:

- ❖ **There is no “right” shoe for everyone with knee OA**
- ❖ **Flat, flexible shoes can decrease the stress on knees for some people.**
- ❖ **Some people benefit from more supportive, stable shoes.**
- ❖ **High heels are bad for your feet and other joints.**
- ❖ **Clogs without backs are also bad for people with knee OA.**

Some people with OA may also benefit from:

- ✓ **Shoe insoles**
- ✓ **Custom made shoe inserts (orthotics)**
- ✓ **Heel wedges**

A physical therapist or podiatrist can recommend the best type of shoe for you.

PHYSICAL ACTIVITY AND OSTEOARTHRITIS

Regular physical activity can:

- **Improve physical function**
- **Lessen joint pain**
- **Strengthen muscles**
- **Increase energy**

Here are some tips for exercising with OA:

- **Choose low impact activity like walking, biking or swimming.**
- **Try to do a combination of:**
  - **Aerobic exercises (like walking): most days of the week.**
  - **Stretching exercises: every day**
  - **Muscle strengthening exercises: 2-3 times per week**
- **Exercise during times of day when you usually have the least pain.**
- **Try icing your knees after exercise to reduce pain and swelling.**
- **Try local exercise classes for people with arthritis.**
- **The Arthritis Foundation (www.arthritis.org) has more information and example exercises.**
- **A physical therapist can help you with a personalized exercise program.**

HEALTHY WEIGHT AND OSTEOARTHRITIS

Having a healthy weight is one of the most important things you can do to take care of your joints.

Losing just 1 pound reduces the weight on your knees by 4 pounds during walking!

Losing 10 pounds makes many people with OA feel better.

Here are some tips for healthy eating:

- ✓ **Avoid sweet tea, sodas and other sugared drinks. They have a lot of calories!**
- ✓ **Avoid saturated fats and trans-fats.**
- ✓ **Choose fruits and green vegetables often.**
- ✓ **Ask others to help you eat healthily.**

The VA MOVE! Program can help you with healthy eating and weight loss. Your doctor can refer you to this program.

PROTECTING YOUR JOINTS

When you have osteoarthritis, it is important to take care of your joints. This can reduce pain and risk of injury. Here are some simple tips:

- **Keep moving! When you stay in one position for too long, your joints can get stiff and sore.**
- **Spread the load across more than one joint. Don't put all the stress on a painful joint.**
- **Take breaks from activity when your joints hurt.**
- **Use railings for support when going up or down stairs.**
- **Ask your doctor or a physical therapist about a cane or walking stick if your knees hurt a lot when you walk.**
- **Other tools can help you with tasks that hurt your joints. Ask a physical therapist or check the Arthritis Foundation website (www.arthritis.org) for ideas.**

COPING WITH OSTEOARTHRITIS PAIN

Pain can affect many aspects of daily life. Here are some important tips for coping with osteoarthritis pain:

- Talk with family or friends about how your pain affects you. Ask them for help when you need it.
- Check for muscle tension around your joints. This can increase pain. Focus on relaxing those muscles when they are tense.
- Pace your activities so you don't "overdo it."
- During painful activities (like going up stairs), it can help to distract yourself. You can think about a calm or happy image.
- Watch for unhelpful thoughts, like "I can't do anything because of my knee pain." These thoughts can work against you. Try to replace these thoughts with more helpful ones, like "I can do my best to stay active even though I have knee pain."
- Take time to do activities you enjoy!

KNEE REPLACEMENT SURGERY

Knee replacement surgery can help with pain and function when joints are badly damaged. It is usually done when people have a lot of pain after trying other treatments.

Here are some things to know about this surgery:

- ❖ Many people with knee OA have good improvements in pain and function after surgery.
- ❖ There are also some risks, as with any surgery.
- ❖ You will need some recovery time and rehabilitation after surgery.
- ❖ People do better with this surgery when they are healthy and strong. If you are thinking about this surgery:
- ❖ Do your best to get to a **healthy weight**.
- ❖ **Exercise regularly**. This can be hard if you have a lot of knee pain. See if a physical therapist if you need help.

Your doctor or an orthopedic surgeon can tell you more about the benefits and risks of knee replacement surgery. They can help you decide if surgery is right for you.

COMPLEMENTARY AND ALTERNATIVE THERAPIES

Some possible osteoarthritis (OA) treatments are not part of "standard" medical care. We don't know much about how well these treatments work.

These treatments seem like they may be helpful for some people with OA:

- ✓ **Massage therapy**
- ✓ **Acupuncture**
- ✓ **Tai Chi**

- **Glucosamine and chrondroitin are often used by people with OA.**
- **Experts are still not yet sure how well these work for OA.**
- **Talk with your doctor if you want more information about these supplements.**

Here are some key tips about complementary and alternative therapies:

- ➢ **They should not replace standard medical care**
- ➢ **Dietary supplements can interact with medications you are taking**
- ➢ **You should talk with your doctor before trying any complementary treatments**

OSTEOARTHRITIS AND SLEEP

Osteoarthritis (OA) pain can make it hard to sleep. Sleep can also make your pain worse.

If you have difficulty sleeping, here are some tips:

- **Don't eat a lot right before bed**
- **Don't drink caffeine in the evening**
- **Try to stick to a regular sleep schedule**
- **Consider taking pain medications at night**
- **Use a firm mattress that supports your joints**
- **Put pillows under sore joints to support them**
- **If you are overweight, this increases the risk of "sleep apnea" (pauses in breathing during sleep).**

Talk with your doctor about:

- **A pain medication schedule that may help with night pain**
- **Other medications that may help you sleep**
- **Whether sleep apnea may be a problem for you**

OSTEOARTHRITIS AND MENTAL HEALTH

Pain and other difficulties from osteoarthritis (OA) can lead people to feel:

- **Stressed**
- **Frustrated**
- **Anxious**
- **Sad**
- **Depressed**
- **Lonely**

If you feel any of these things you are not alone.
There are things you can do to help:

- ✓ **Talk with family or friends about how you are feeling. Others can help you with the effects of OA on your life.**
- ✓ **Take time out to relax and refresh yourself when you need it.**
- ✓ **Keep active. Exercise can help with feelings of anxiety and depression.**
- ✓ **Talk with your doctor if you feel depressed, especially for two weeks or more. There are treatments that can help you.**
- ✓ **Your doctor can also refer you to a counselor who can help you cope with pain and other effects of OA.**

OSTEOARTHRITIS AND FATIGUE

Many people with osteoarthritis (OA) feel fatigue.

Some reasons for this may be:

- **Poor sleep**
- **Daily tasks take more effort when you have pain**
- **Medication side effects**
- **Stress or depression**

Here are some tips for fighting fatigue:

- **Take breaks between activities. Listen to your body, and stop before you are too tired.**
- **Try using a cane or stick if walking makes you sore or very tired.**
- **Get enough sleep. If you are not sleeping well, tell your doctor.**
- **Keep active. Regular activity can actually help reduce fatigue.**
- **Eat well. Healthy foods give your body the energy it needs to do your daily activities.**
